# Risk factors of acute coagulation dysfunction after aneurysmal subarachnoid hemorrhage

**DOI:** 10.1186/s41016-018-0135-6

**Published:** 2018-10-08

**Authors:** Guo-Rong Chen, Pei-Sen Yao, Chu-Bin Liu, Huang-Cheng Shang-Guan, Shu-Fa Zheng, Liang-Hong Yu, Yuan-Xiang Lin, Zhang-Ya Lin, De-Zhi Kang

**Affiliations:** 1 Department of Neurosurgery, The First Affiliated Hospital of Fujian Medical University, NO. 20 Chazhong Road, Taijiang District, Fuzhou City, 350004 Fujian China; 2 Department of Neurosurgery, The Second Affiliated Hospital of Fujian Medical University, Quanzhou City, 362000 Fujian China

**Keywords:** Risk factors, Coagulation dysfunction, Aneurysmal subarachnoid hemorrhage

## Abstract

**Background:**

Although coagulopathy have been proved to be a contributor to a poor outcome of aneurysmal subarachnoid hemorrhage (aSAH), the risk factors for triggering coagulation abnormalities have not been studied after aneurysm clipping.

**Methods:**

We investigated risk factors of coagulopathy and analyzed the relationship between acute coagulopathy and outcome after aneurysm clipping. The clinical data of 137 patients with ruptured CA admitted to our institution was collected and retrospectively reviewed. Patient demographic data (age, sex), smoking, alcohol use, hypertension, diabetes, Hunt-Hess grade, Fisher grade, operation time, intraoperative total infusion volume, intraoperative blood loss, intraoperative transfusion, intraoperative hemostatic drug treatment, calcium reduction (preoperative free calcium concentration–postoperative free calcium concentration) were recorded. Coagulation was assessed within 24 h. Postoperative hemorrhage and infarction, deep venous thrombosis (DVT), and mortality were analyzed.

**Results:**

Coagulopathy was detected in a total of 51 cases (group I), while not in 86 cases (group II). Univariable analysis demonstrated that age, smoking, alcohol use, intraoperative total infusion volume, intraoperative blood loss, intraoperative transfusion, and calcium reduction (≥ 1.2 mg/dl**)** were related to coagulopathy. Non-conditional logistic regression analysis showed that age [OR, 1.037 (95% CI, 1.001–1.074); *p* = 0.045] and calcium reduction (≥ 1.2 mg/dl) [OR, 5.509 (95% CI, 1.900–15.971); *p* = 0.002] were considered as the risk factors for coagulopathy. Hunt-Hess grade [OR, 2.641 (95% CI, 1.079–6.331); *p* = 0.033] and operation time [OR, 0.107 (95% CI, 1.012–0.928); *p* = 0.043] were considered as the risk factors for hypocoagulopathy. There were 6 cases (11.7%) with cerebral infarction in group I, while 6 cases (6.98%) in group II (*χ*^2^ = 0.918, *p* = 0.338). There were 4 cases (7.84%) with rebleeding in group I, while 5 cases (5.81%) in group II (*χ*^2^ = 0.215, *p* = 0.643). The mortality was 9.80% (5/51) in group I, while 1.16% (1/86) in group II (*χ*^2^ = 5.708, *p* = 0.017). DVT was not detected in all cases.

**Conclusions:**

In conclusion, age (≥ 65 years) and calcium reduction (≥ 1.2 mg/dl) were considered as the risk factors for coagulopathy and have been proved to be associated with higher mortality after aneurysm clipping.

## Background

Aneurysmal subarachnoid hemorrhage (aSAH) accounts for 3–5% of strokes, which is serious harmful to human health for its high mortality and morbidity [1, 2]. Although neurosurgical clipping following aSAH prevents rerupture of aneurysms, reduces the mortality and cognition, and improves the Glasgow Coma Scale (GCS), neurological deficits were detected after intracranial aneurysm clipping [3–7]. And it is known that postoperative hemorrhagic or ischemic injury caused by systematic coagulopathy was closely related to these complications [4, 8, 9].

Previous studies indicated the older patients with coagulation abnormalities are more likely to develop progressive hemorrhagic injury in traumatic brain injury (TBI) patients, including subarachnoid hemorrhage, epidural hematoma, and subdural hematoma [4]. Elevated serum fibrinogen degradation product (FDP) was associated with intracranial hemorrhage and mass effect following TBI [10]. In addition, coagulative/fibrinolytic abnormalities are the frequently cause for intracranial re-hemorrhage [11]. Hypercoagulability has been proved to be associated with cerebral arterial ischemic stroke [12]. Giroud et al. demonstrated that significant activation of the coagulation was observed in the patients with cortical infarction [13].

Furthermore, coagulopathy after TBI is a powerful predictor related to poor outcome [14], including higher in-hospital mortality, increased incidence of DVT and worse long-term functional outcomes [4, 15]. It was revealed that coagulation abnormalities were related to poor outcome in the acute stage of ruptured intracranial aneurysm [16, 17]. Ettinger found that patients with fibrinogen values over 400 mg/100 ml shortly after subarachnoid hemorrhage had worse prognosis [18].

Although coagulopathy has been proved to be a contributor to the poor outcome of aSAH, the risk factors for triggering coagulation abnormalities have not been clarified after neurosurgical clipping. In the study, we reviewed risk factors of acute coagulopathy and analyzed the relationship between acute coagulopathy and the outcome in the aSAH patients after surgical clipping.

## Methods

### Patients

This prospective study was performed in the First Affiliated Hospital of Fujian Medical University. The study was approved by the ethics committee of the First Affiliated Hospital of Fujian Medical University. All patients provided written informed consent.

Patients were collected in the trial if the following conditions were met: (1) Subarachnoid hemorrhages were diagnosed by computed tomography (CT). Cerebral aneurysms were diagnosed by computerized tomography angiography (CTA) and/or digital subtraction angiography (DSA). (2) Patients underwent a minimally invasive pterional or supraorbital keyhole approach after hemorrhage by the same surgeon. (3) Patients with preoperative coagulation abnormalities, hematological disorders, ongoing anticoagulant treatment, active alcohol or drug abuse, or malignant tumor were excluded.

### Assessment of coagulopathy

We reviewed the clinical and biological correlates of coagulopathy in a large series of patients undergoing aneurysmal clipping. Coagulation meeting any one of following conditions was defined as coagulopathy: (1) fibrinogen level less than 2 mg/dl or greater than 4 mg/dl; (2) prolongation or shortening of prothrombin time (PT) greater than 3 s; (3) prolongation or shortening of activated partial thromboplastin time (APTT) greater than 10 s; (4) prolongation or shortening of thrombin time (TT) greater than 3 s; (5) abnormalities of D-dimmer were detected; (6) a positive test plasma protamine paracoagulation test was detected. Coagulation was assessed within 24 h after surgery.

### Analyzed factors and postoperative evaluation

The clinical data of 137 patients with ruptured CA admitted to our institution was collected and retrospectively reviewed. Patient demographic data (age, sex), smoking, alcohol use, hypertension, diabetes, Hunt-Hess grade, Fisher grade, operation time, intraoperative total infusion volume, intraoperative blood loss, intraoperative transfusion, intraoperative hemostatic drug treatment, and calcium reduction (preoperative free calcium concentration–postoperative free calcium concentration) were recorded. Coagulation was assessed within 24 h.

### Statistical analysis

Statistical analysis was performed using SPSS version 18.0 (SPSS Inc., Chicago, IL, USA) for Windows. The chi-square (*χ*^2^) test was used for categorical data, and multivariate analysis was carried out while there was statistical difference firstly analyzed by one-way ANOVA. Non-conditional logistic regression analysis was performed to identify the effects of multi factors on prognosis. A *P* value less than 0.05 was considered as statistically significant. Basic clinical characteristics of patients are shown on Table 1. Univariable logistic regression analyses included all variables’ significance level at *p* < 0.15.

**Table 1:** Basic clinical characteristics of patients with aneurismal clipping

General information	Normal 86 cases	Coagulopathy 51 cases	*χ*^2^ value	*P* value
Age				
< 65 years	77	37	6.612	0.010
≥ 65 years	9	14		
Sex				
Male	39	20	0.491	0.483
Female	47	31		
Smoking				
Yes	16	5	6.563	0.010
No	70	81		
Alcohol				
Yes	8	1	5.745	0.017
No	78	85		
Hypertension				
Yes	41	19	0.918	0.338
No	49	32		
Diabetes				
Yes	3	3	0.438	0.508
No	83	48		
Hunt-Hess grade				
I–III	73	42	0.152	0.697
IV–V	13	9		
Fisher grade				
1–2	46	31	0.692	0.405
3–4	40	20		
Operation time				
≤ 3 h	41	29	1.082	0.298
> 3 h	45	22		
Intraoperative total infusion volume				
≤ 2000 ml	14	3	47.300	0.000
2000–4000 ml	61	37		
≥ 4000 ml	11	11		
Intraoperative blood loss				
≤ 300 ml	50	28	20.470	0.000
300–600 ml	13	8		
≥ 600 ml	23	15		
Intraoperative transfusion				
Yes	7	10	3.874	0.049
No	79	41		
Intraoperative hemostatic drug				
Yes	13	9	0.152	0.697
No	73	42		
Calcium reduction				
< 1.20 mg/dl	10	17	60.223	0.000
0–1.20 mg/dl	65	31		
≥ 1.20 mg/dl	11	3		

The following risk factors for coagulopathy were entered in the univariable analysis: age (greater than 65 years or less than 65 years), smoking, alcohol use, intraoperative total infusion volume, intraoperative blood loss, intraoperative transfusion, and calcium reduction (≥1.2 mg/dl).

## Results

A consecutive series of 137 patients (range 10–86 years, mean age 53.60 ± 11.38 years, 59 males and 78 females) were collected between January 2012 and January 2014 in this prospective study. There were a total of 252 aneurysms: 13 located at anterior cerebral artery (ACA), 32 at the posterior communicating artery (PCoA), 68 at the middle cerebral artery (MCA), 69 at the anterior communicating artery (ACoA), 52 at the internal carotid artery (ICA), 3 at the posterior cerebral artery (PCA), 4 at the ophthalmic artery, 7 at the anterior choroidal artery, 1 at the basilar artery, and 2 at vertebral artery. A total of 33 patients were presenting with multiple aneurysms. There were 17 patients presenting with Hunt-Hess grade I, 64 with grade II, 33 with grade III, 15 with grade IV, and 7 with grade V.

Basic clinical characteristics of patients are shown on Table 1. Coagulopathy was detected in a total of 51 cases (group I), while not in 86 cases (group II). Univariable analysis demonstrated that age, smoking, alcohol use, intraoperative total infusion volume, intraoperative blood loss, intraoperative transfusion, and calcium reduction (≥ 1.2 mg/dl**)** were related to coagulopathy. Non-conditional logistic regression analysis showed that age [odd ratio (OR), 1.037 (95% confidence interval, CI, 1.001–1.074); *p* = 0.045] and calcium reduction (≥ 1.2 mg/dl) [OR, 5.509 (95% CI, 1.900–15.971); *p* = 0.002] were considered as the risk factors for coagulopathy (Table 2).

**Table 2:** Multivariate logistic regression analysis of acute coagulopathy risk factors

Variables	Exp (B)	95% Exp (B)		*P* value
		Lower limit	Upper limit	
Age	1.037	1.001	1.074	0.045
Smoking	1.008	0.272	3.736	0.991
Alcohol	0.199	0.014	2.848	0.234
Intraoperative net fluid intake	1.000	1.000	1.001	0.571
Intraoperative blood loss	0.999	0.997	1.001	0268
Intraoperative transfusion	4.163	0.910	19.032	0.660
Calcium reduction (≥ 1.2 mg/dl)	5.509	1.900	15.971	0.002

The clinical characteristics of patients with hypocoagulopathy and hypercoagulopathy are shown in Table 3. Hypocoagulopathy was detected in a total of 40 cases (subgroup I), while hypercoagulopathy in 11 cases (subgroup II). Univariable analysis demonstrated that Hunt-Hess grade, operation time, intraoperative total infusion volume, intraoperative blood loss, and calcium reduction were related to coagulopathy. Non-conditional logistic regression analysis showed that Hunt-Hess grade [OR, 2.641 (95% CI, 1.079–6.331); *p* = 0.033] and operation time [OR, 0.107 (95% CI, 1.012–0.928); *p* = 0.043] were considered as the risk factors for hypocoagulopathy (Table 4).

**Table 3:** Basic clinical characteristics of patients with coagulation dysfunction

General information	Hypocoagulopathy 40 cases	Hypercoagulopathy 11 cases	*χ*^2^ value	*P* value
Age				
< 65 years	28	9	0.605	0.437
≥ 65 years	12	2		
Sex				
Male	14	6	1.383	0.240
Female	26	5		
Smoking				
Yes	3	2	1.113	0.291
No	37	9		
Alcohol				
Yes	1	0	0.281	0.596
No	39	11		
Hypertension				
Yes	14	5	0.403	0.525
No	26	6		
Diabetes				
Yes	2	1	0.261	0.610
No	38	10		
Hunt-Hess grade				
I–III	35	7	3.381	0.066
IV–V	5	4		
Fisher grade				
1–2	18	2	2.603	0.107
3–4	22	9		
Operation time				
≤ 3 h	14	9	5.987	0.014
> 3 h	26	3		
Intraoperative total infusion volume				
≤ 2000 ml	4	3	15.321	0.000
2000–4000 ml	28	4		
≥ 4000 ml	8	4		
Intraoperative blood loss				
≤ 300 ml	7	8	6.394	0.041
300–600 ml	20	1		
≥ 600 ml	13	2		
Intraoperative transfusion				
Yes	9	1	0.984	0.321
No	31	10		
Intraoperative hemostatic drug				
Yes	6	3	0.894	0.344
No	34	8		
Calcium reduction				
≤ 0 mg/dl	2	1	39.707	0.000
0–1.20 mg/dl	21	10		
≥ 1.20 mg/dl	17	0

**Table 4:** Multivariate logistic regression analysis of hypocoagulopathy risk factors

Variables	Exp (B)	95% Exp (B)		*P* value
		Lower limit	Upper limit	
Hunt-Hess grade	2.614	1.079	6.331	0.033
Operation time	0.107	0.012	0.928	0.043
Intraoperative total infusion	1.001	1.000	1.003	0.054
Intraoperative blood loss	0.997	0.992	1.002	0.209
Calcium reduction	2.751	0.410	18.478	0.298

There were 6 cases (11.7%) with cerebral infarction in group I, while 6 cases (6.98%) in group II (*χ*^2^ = 0.918, *p* = 0.338). There were 4 cases (7.84%) with rebleeding in group I, while 5 cases (5.81%) in group II (*χ*^2^ = 0.215, *p* = 0.643). The mortality was 9.80% (5/51) in group I, while 1.16% (1/86) in group II (*χ*^2^ = 5.708, *p* = 0.017). DVT was not detected in all cases.

## Discussion

Although neurosurgical clipping following aSAH prevents aneurysm rerupture, neurological deficit, which was related to postoperative hemorrhagic or ischemic injury, was often detected after intracranial aneurysm clipping [3–7]. Previous studies indicated that coagulopathy was positively associated with the risk of hemorrhagic or ischemic brain injury after TBI [4, 8, 9, 19]. However, no studies paid attention to the risk factors for triggering coagulation abnormalities after aneurysm clipping. In the study, age, smoking, alcohol use, intraoperative total infusion volume, intraoperative blood loss, intraoperative transfusion, and calcium reduction (≥ 1.2 mg/dl**)** were related to coagulopathy. However, non-conditional logistic regression showed that age and calcium reduction (≥ 1.2 mg/dl) were considered as the risk factors for coagulopathy.

Operation possesses a significant risk of neurological dysfunction and death in patients > 65 years of age with unruptured intracranial aneurysms [20] . Increasing age is associated with higher neurological morbidity and mortality after pipeline embolization of intracranial aneurysms [21]. And in the study, age more than 65 years was an independent risk factor for coagulopathy and associated with higher mortality after aneurysm clipping. Previous studies indicated that higher tissue plasminogen activator (tPA) and lower activity of plasminogen activator inhibitor (PAI) were detected in older patients. And after surgery, lower plasma concentrations of PAI-1 preoperatively leads to higher plasma levels of D-dimer in association with coagulopathy [22]. However, increasing age was not related to higher postoperative hemorrhage and infarction, which was consistent with our findings.

Ionized calcium was defined as coagulation factors IV and played a critical role in the activity of coagulation. Hypocalcemia is a common finding in critically ill patients, especially in critically ill surgical patients, and possesses the prognostic value of poor outcome [23, 24]. Reduction of ionized calcium more than 1.2 mg/dl was associated with hypocoagulopathy and the poor outcome of aSAH patient after aneurysm clipping in our study. However, our findings indicate that calcium reduction is not associated with the hypercoagulopathy or hypocoagulopathy in the aSAH patients.

Here, it is shown that Hunt-Hess grade and operation time is considered as the risk factors for hypocoagulopathy, which has not been reported previously. We deduce that poor Hunt-Hess grade correlates with increased intracranial pressure [25], which might prevent intraoperative exposure of the cerebral aneurysm, prolong the operation time (“skin-to-skin”), and increase iatrogenic injury to brain tissue, the latter would cause the release of tissue factor, and then initiate exogenous coagulation pathway, finally lead to coagulopathy [26]. In addition, the acute brain injury following aneurysm rupture in poor condition (Hunt-Hess grade IV–V) is more serious than good condition (I–III), which will also result in the release of tissue factor. Certainly, these conclusions are drawn from a retrospective study, and it cannot be fully confirmed whether operation time is a risk factor for hypocoagulopathy; our findings should be verified in a larger prospective study.

## Conclusions

In conclusion, age (≥ 65 years) and calcium reduction (≥ 1.2 mg/dl) were considered as the risk factors for coagulopathy and have been proved to be associated with poor outcome after aneurismal clipping. However, postoperative coagulopathy was not related to postoperative hemorrhage and infarction. As our conclusions were drawn from a single institution experience, a larger sample is needed in order to verify our findings.

## Abbreviations

ACA: Anterior cerebral artery
ACoA: Anterior communicating artery
APTT: Activated partial thromboplastin time
aSAH: Aneurysmal SAH
CT: Computed tomography
CTA: CT angiography
DSA: Digital subtraction angiography
DVT: Deep venous thrombosis
FDP: Fibrinogen degradation product
GCS: Glasgow Coma Scale
ICA: Internal carotid artery
MCA: Middle cerebral artery
PAI: Plasminogen activator inhibitor
PCA: Posterior cerebral artery
PCoA: Posterior communicating artery
PT: Prothrombin time
SAH: Subarachnoid hemorrhage
TBI: Traumatic brain injury
tPA: Tissue plasminogen activator
TT: Thrombin time
